# Development of a Biocrystallisation Assay for Examining Effects of Homeopathic Preparations Using Cress Seedlings

**DOI:** 10.1155/2012/125945

**Published:** 2012-08-27

**Authors:** S. Baumgartner, P. Doesburg, C. Scherr, J.-O. Andersen

**Affiliations:** ^1^Institute of Complementary Medicine KIKOM, University of Bern, Insel-Spital, 3010 Bern, Switzerland; ^2^Hiscia Institute, Kirschweg 9, 4144 Arlesheim, Switzerland; ^3^Center for Integrative Medicine, University of Witten/Herdecke, Gerhard-Kienle-Weg 4, 58313 Herdecke, Germany; ^4^Louis Bolk Institute, Hoofdstraat 24, 3972 LA Driebergen, The Netherlands; ^5^Crystal Lab, Landgoed Roepaen, Kleefseweg 9, 6595 NK Ottersum, The Netherlands; ^6^Biodynamic Research Association Denmark, Landsbyvaenget 7, Herskind, 8464 Galten, Denmark

## Abstract

A major challenge in basic research into homeopathic potentisation is to develop bioassays that yield consistent results. We evaluated the potential of a seedling-biocrystallisation method. Cress seeds (*Lepidium sativum* L.) germinated and grew for 4 days * in vitro* in *Stannum metallicum* 30x or water 30x in blinded and randomized assignment. 15 experiments were performed at two laboratories. CuCl_2_-biocrystallisation of seedlings extracted in the homeopathic preparations was performed on circular glass plates. Resulting biocrystallograms were analysed by computerized textural image analysis. All texture analysis variables analysed yielded significant results for the homeopathic treatment; thus the texture of the biocrystallograms of homeopathically treated cress exhibited specific characteristics. Two texture analysis variables yielded differences between the internal replicates, most probably due to a processing order effect. There were only minor differences between the results of the two laboratories. The biocrystallisation method seems to be a promising complementary outcome measure for plant bioassays investigating effects of homeopathic preparations.

## 1. Introduction

A major challenge in homeopathic basic research is the development of well-defined bioassays that generate reproducible evidence for specific effects of homeopathic preparations [1]. During recent years various studies have been reported that successfully used healthy plants (e.g., wheat, peas, duckweed) as test organisms [2]. Amongst those, test systems based on seedlings have various advantages: seedlings are easily handled, they can be grown in great numbers thereby enhancing the possibility of observing significant differences between treatments, and the growth process may be recorded nondestructively, allowing the option of further investigations (e.g., chemical analysis) at the end of an experiment.

**Figure 1:**
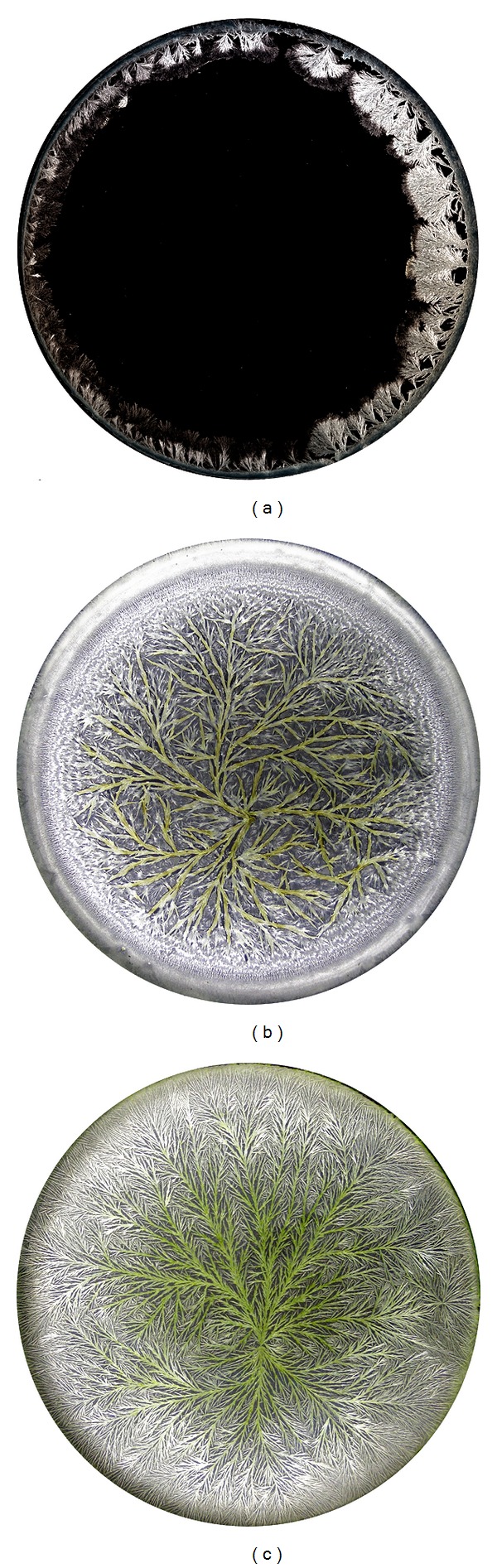
Biocrystallograms on circular glass plates (90 mm diameter), produced on the basis of (a) watery CuCl_2_ solution (150 mg per glass plate), without additive; (b) watery extract of barley flour (60 mg extract 10% (w/v)/120 mg CuCl_2_/6.0 mL volume); (c) watery extract of cress seedlings germinated in distilled water (310 mg extract 5% (w/v)/150 mg CuCl_2_/6.1 mL volume).

The picture-developing CuCl_2_ crystallisation method, also termed biocrystallisation, may be integrated into a seedling test system for homeopathic preparations. The basis of this method is the phenomenon that when crystallising watery solutions of dihydrate CuCl_2_ in the presence of organic additives (juices/extracts), reproducible dendritic structures can be observed [3–6]. The experimental procedure is thus based on evaporation/crystallisation of watery solutions of dihydrate CuCl_2_ and the organic additive in question on circular glass plates, leading to three-dimensional structures of CuCl_2_ crystals (biocrystallograms, see Figure 1). These structures are concentration specific as well as sample and treatment specific [7].

The method has been used as a complementary (“holistic”) method to characterize qualitative aspects of various additives and also to investigate the effects of farming systems and fertilisation treatments on agricultural/horticultural crop samples [8–11].

During recent years several experimental aspects of the biocrystallisation method have been standardised and validated within the so-called Triangle consortium (three laboratories from Germany, The Netherlands, and Denmark; the latter two in the following referred to as LBI and BRAD, resp.), including lab procedures, crystallisation equipment and techniques, visual scoring of morphological criteria observed in the crystal structures, as well as computer-based textural image analysis based on spatial variation in pixel grey values (grey level cooccurrence matrices approach) [7, 12–15]. The image analysis not only adds credibility to the evaluation, but also introduces the possibility to analyse large datasets generated on the basis of crystallisation images.

Previously Selawry [16] applied the biocrystallisation method as tool to investigate the effects of homeopathic preparations. In these studies, germinating grain and legume seeds were treated with various metal potencies. Corresponding extracts were reported to show both sample- and potency-specific macro- and microscopic dendritic structures. However, major reservations must be made towards the results and conclusions of these studies, due to a lack of experimental details on the applied germination, crystallization, and evaluation procedures.

The goals of the present methodological and exploratory study were the following:

- to develop a test system for examining effects of homeopathic preparations, based on biocrystallograms of extracts of cress seedlings germinating within homeopathic preparations and being extracted with homeopathic preparations, and to report it according to current standards [17];
- to apply the test system at two independent laboratories (LBI, BRAD) in repeated experiments, based on a comparison of two 30x preparations;
- to determine promising primary outcome parameters for further use in future confirmatory experiments.

## 2. Materials and Methods

### 2.1. General Experimental Procedure

Cress seedlings germinated and grew for approximately 96 h in hanging plastic bags on chromatography paper that had been soaked with either aqueous homeopathic preparations or control solutions. A watery extract was produced based on a filtrate of mashed seedlings that were crushed and extracted in the corresponding homeopathic preparation or control solution. This extract was mixed with dihydrate CuCl_2_ solution and pipetted on levelled glass plates. Evaporation under standardized conditions led to a formation of CuCl_2_ crystals on the glass surface. The resulting biocrystallograms were evaluated by means of visual evaluation as well as computerised image analysis. Experiments were randomized and blinded, and the code was revealed only after completion of the evaluation. 

### 2.2. General Experimental Design

#### 2.2.1. Screening Experiments

In a number of screening experiments in both laboratories, eight preparations (encoded A–H) were examined in blind. The eight preparations, produced and supplied by Allergica (Silkeborg, Denmark), comprised seven 30x preparations (*Aurum metallicum, Stannum metallicum, Cuprum metallicum,* copper sulfate, gibberellic acid, lactose, and distilled water) and additionally nonpotentised distilled water. A preselection of four out of these eight preparations was done, based on the observation that these four preparations seemed to exhibit recurring differences in morphological characteristics in the biocrystallograms produced, being in line with some texture analysis parameters. After some further blinded screening experiments, two out of these four preparations (codes B and G) were selected to be investigated in a series of repeated standardized experiments (see Section 2.2.2).

#### 2.2.2. Main Experiments

**Figure 2:**
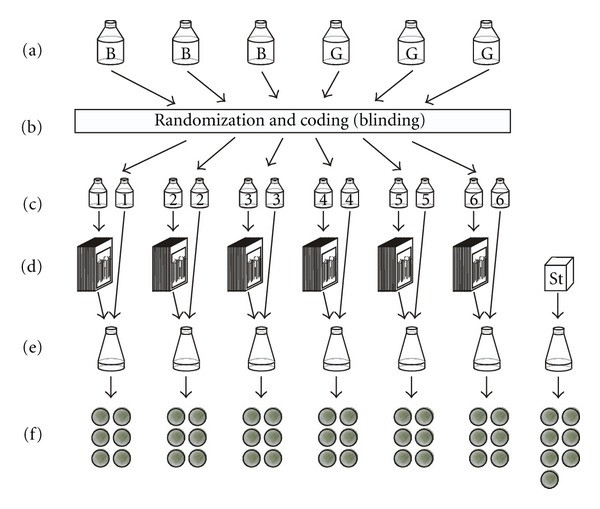
General design of one main experiment. (a) The coded homeopathic preparations B and G (corresponding to *Stannum metallicum* 30x and water 30x) had been prepared in triplicate. (b) The six preparations were randomized and coded with numbers 1–6. (c) The preparations were filled in two separate bottles each. (d) The content of one of these bottles was used to cultivate the cress seedlings (soaking 10 filter papers in plastic bags for each of the six experimental conditions). (e) An aqueous extract was prepared from crushed seedlings after a 96 h growth period, extracted in homeopathic solution using the 2nd coded bottle. (f) For each experimental condition, six biocrystallograms were prepared. In addition, seven biocrystallograms were prepared using an open (noncoded) internal standard “St” (freeze-dried wheat).

The two selected preparations—still coded as B and G—were investigated in randomized and coded (“blind”) allocation in 15 independent experiments in total, thereof seven at LBI and eight at BRAD. In each experiment, six cress extracts corresponding to six experimental parameters (the two preparations B and G prepared in triplicate each) were examined in sixfold glass plate replicates yielding 36 biocrystallograms (Figure 2) and in addition seven biocrystallograms of a reference compound (freeze-dried wheat), thereby applying the crystallisation apparatus capacity of 43 biocrystallograms per day.

This experimental design allowed controlling reproducibility as well as experimental stability at the same time. Reproducibility could be examined between laboratories, between experiments (by comparing the results as a function of individual experiments), and within experiments (by comparing the results as a function of the three internal replicates). Since one of the preparations was water 30x (prepared in triplicate), the Systematic Negative Control approach [18] was incorporated into the experimental design in modified form.

### 2.3. Homeopathic Preparations

All potency and control preparations were produced and supplied by the company Allergica (Silkeborg, Denmark). For homeopathic potentisation, a given substance was repeatedly diluted and succussed, first in the solid phase (using lactose as dilution medium) and subsequently in the liquid phase (using distilled water). In the present case the dilution ratio was 1 : 9 for every solid and liquid potentisation step, and the dilution/succussion procedure was repeated 30x yielding a nominal dilution of 10^−30^ of substances potentised.

For the screening experiments, eight different preparations (*Aurum met.* 30x, *Stannum met.* 30x, *Cuprum met.* 30x, copper sulfate 30x, gibberellic acid 30x, lactose 30x, distilled water 30x, and nonpotentised distilled water) were produced. Water-insoluble compounds (*Aurum met.*, *Stannum met.*, *Cuprum met.*) were triturated in lactose up to 4x and then further potentised in distilled water up to 30x. All other substances were dissolved in distilled water and potentised up to 30x. Trituration was effectuated by hand using a porcelain mortar and pestle, lasting 60 minutes for each potentisation level. Liquid potentisation was performed by moving test tubes (100 × 24 mm, silicone stopper; Hounisens Laboratorieudstyr, Risskov, Denmark) in lemniscate movements by hand (standard production procedure in the company Allergica). Homeopathic preparations and controls were coded A–G, filled in 5 litre plastic containers (HDPE polyethylene; Grahtwol, Karlslunde, Denmark), and supplied to the two laboratories.

Sources for the materials used were as follows: *Aurum metallicum* (Weleda, Schwäbisch-Gmünd, Germany), *Stannum metallicum* (Weleda, Schwäbisch-Gmünd, Germany), *Cuprum metallicum* (Weleda, Schwäbisch-Gmünd, Germany), copper sulfate (CuSO_4_ × 5H_2_O; Fluka, Buchs, Switzerland), gibberellic acid (S4171064 636, 8.14464.0001; Merck, Hohenbrunn, Germany), and lactose (Nomeco, Copenhagen, Denmark). Distilled water for potentisation was supplied by Nomeco (Copenhagen, Denmark).

For the main experiments, two aqueous 28x potency preparations (*Stannum metallicum* and water) were produced as described above. From each of these two 28x potency preparations subsequently a total of 48 independent 30x preparations were produced. Thus in total 96 30x preparations were produced for 2 labs allowing 8 experiments to be performed, each consisting of 3 replicates of the 2 preparations (2 × 8 × 3 × 2 = 96). Each preparation was supplied as two portions of 50 mL, intended for germination and extraction fluid, respectively, and specified as I and II. Out of the 8 experiments available for each lab, 7 and 8 were subsequently performed at LBI and BRAD, respectively.

### 2.4. Coding (Blinding), Randomization and Decoding

The 8 samples for the screening experiments were manually coded A–G by Allergica. This code was revealed to the first author (not being involved in any experimental work) after performance of the screening experiments in order to decide which of these preparations should be included in the main experiments, based on the results of the screening experiments performed at BRAD and LBI in blind. Only after completion of the visual evaluation and the computerised image analysis of the main experiments, the code holder (SB) revealed the code to the two laboratories (JOA at BRAD and PD at LBI), showing that B represented *Stannum metallicum* 30x and G represented distilled water 30x.

The 96 samples for the main experiments were also manually randomized and coded by Allergica. The 6 potency preparations per experiment were randomised and subsequently manually coded 1–6, 7–12*⋯*91–96, whereby 1–48 and 49–96 were shipped to LBI and BRAD, respectively. After completion of the visual evaluation and the computerised image analysis of the main experiments, this code was revealed to the first author for the statistical analysis and subsequently forwarded to LBI and BRAD.

### 2.5. Seed Germination and Growth

The applied germination procedure was a modification of the procedure developed by Baumgartner and Flückiger [19]. Garden cress seeds (*Lepidium sativum* L.; Bingenheimer Saatgut AG, Echzell-Bingenheim, Germany; article no. G250, Kresse Einfache) were used. Seeds that were damaged and/or not showing characteristics of fully developed seeds were discarded. Germination took place on filter papers (blotting paper 151B; 85 × 140 mm; 87 g/m^2^; thickness 0.17 mm; Frisenette, Knebel, Denmark), placed in plastic bags (MiNiGRiP Colorline; type 11–16; 100 × 150 × 0.05 mm; JOKA Plastic-Emballage A/S, Holte, Denmark). The fibre direction of the filters was positioned downwards and germination was performed on the “smooth” side. A volume of 3.00 mL potency preparation from bottle I of the preparation was pipetted onto each filter. A total of 10 filters were used per preparation. A code was written on each bag specifying the experiment (1–8), preparation number (1–48 for LBI, 49–96 for BRAD), and bag replicate (1–10). When the solution was absorbed evenly over the filter, 16 seeds were placed on the filter. A minimum access of air into the bags was arranged by manually expanding the upper closing area of the bags at both sides.

The bags were placed in a germination box (polyethylene; 200 × 315 × 450 mm), hanging vertically from stainless steel rods (diameter 3 mm) free from contact with the bottom and the lid. The bags were positioned on the rods according to the processing order of the 6 potency preparations (i.e., no. 1–3 on the first rod and no. 4–6 on the other). Bags belonging to the different preparations were separated by a plastic bag containing a filter paper covered with double layers of aluminium foil. Similarly in the area between the two rows of plastic bags a cardboard covered with a double layer of aluminium foil was placed. The germination box was placed in a heating cabinet at 19°C (±1°C) on a perforated plate. After about 3 h the seeds had developed a mucous sphere, allowing the 16 seeds to be aligned 9 cm from the bottom of the filter at 2-3 mm distance, followed by incubation for approximately 96 h. After this period, seedlings had typically grown to a total shoot and root length of about 9 cm. 

### 2.6. Preparation of Seedlings

After the growth period the plastic bags containing the seedlings were cut open at both length sides, approximately 3 mm from the filter paper and similarly approximately 3 mm above the paper. The front side of the bag was opened, and the upper part of the paper was bent backwards whereby the seedlings were accessible for manual selection of single seedlings. Seedlings with a minimum root length of 6.5 cm were selected from each bag, generally resulting in 8–12 seedlings per bag. Seedlings that deviated in shape or colour, or showed signs of fungal growth, were discarded. The brown seed coats, which generally were separated from the seedlings after germination, were discarded. The seedlings were collected in a Petri dish placed on a balance. The number of seedlings from each bag was noted on a standard form, together with the sum weight of the selected seedlings. For further processing a total of 3.00 g seedlings was applied, made up from on average 10 seedlings per bag, each seedling weighing approximately 0.03 g. The seedlings were placed in a mortar (porcelain; 160 mL; *Ø* = 90 mm; Haldenwanger, Waldkraiburg, Germany) containing 10.0 mL of potency preparation. The mortar was covered with Parafilm M (Pechiney, Menasha, USA) until seedlings from all treatments had been prepared.

The seedlings were crushed by means of a pestle applying diagonal movements for 2 minutes, resulting in no intact leaf or root parts to be observed in the solution. Subsequently lemniscate movements were applied for 1 minute whereby the leaf and root parts were homogenized further. Pestle and mortar were each flushed with 8.50 mL potency, thereby generating a 10% solution on weight basis (3.00 g seedlings; 27.00 mL potency; in total 30.00 g solution). Extraction of the plants in the homeopathic preparations was performed to obtain a maximal differentiation between the experimental groups. The solution was transferred to a wide-necked 100 mL Erlenmeyer flask. Each flask was covered with Parafilm and left standing until all treatments were performed. The solutions were extracted on a horizontal shaker (Heidolph Unimax 2010) at 125 rpm for 45 minutes. The extract was filtered for 3 minutes by means of a nylon filter (pore size 150 *μ*m; 03–150/38; Sefar AG, Heiden, Switzerland) placed in a glass funnel (upper diameter 70 mm, tube length 70 mm, tube diameter 10 mm) placed in a preweighed wide-necked 100 mL Erlenmeyer flask. The weight of the filtrate was noted.

### 2.7. Biocrystallisation

Biocrystallisation was performed at the two labs by means of the Triangle crystallisation techniques and lab procedures described by Kahl [7], during the period June–August 2008. The solutions applied for biocrystallisation were produced on the basis of extract of 310 mg substance (seedlings) per crystallisation plate, in combination with 150 mg of dihydrate CuCl_2_ (copper (II) chloride dihydrate; Merck, article no. 1.02733.1000; pro analysis) from a 5% aqueous solution, hereby generating a volume of 6.1 mL per plate. A total of six biocrystallograms (replicates) were produced per potency preparation per experiment.

The glass plates applied were circular float glass (1st quality float glass; “air side” applied; 100.0 mm diameter; thickness 2.0 mm ± 0.2; Pfaehler GmbH & Co. KG, Gengenbach, Germany). The cleaning of the glass plates, as well as other glass utensils, was performed by means of the following procedure at BRAD: 12 h in 20°C 5% Merck MA 01 Extran bath; thoroughly flushing with tap water; 1 h in 15% nitric acid; 4 consecutive baths of demineralised water, the 4th with maximal conductivity 1.5 *μ*S; flushing with 96% ethanol; drying in air. At LBI, glass plates were cleaned in a Miele desinfector G7735, performing the following three washing steps: neodisher FLA, neodisher Z (both VWR), and deionized water 90°C. The plates were combined with acrylic rings (made from GS acrylic tubes Riacryl; length 35 mm, thickness 5 mm ± 0.5 mm; Broennum Plast, Rodovre, Denmark) to constitute a basin for pipetting the crystallisation solution. The glass plates and the acrylic rings were assembled by means of Vaseline (Prolabo, Fontenay sous Bois, France; article no. 28 908.290).

The 36 crystallisation plates with cress extracts (six treatment groups in 6-fold glass plate replication, see Figure 2) and the seven plate replicates from the freeze-dried reference sample [7] were positioned on the same cell number in the crystallization chamber for each experiment. The two laboratories had different recurring distributions of the 43 available positions among the 6 + 1 preparations.

Steady-state condition for temperature was regulated at 30°C (±1°C) at 14.4 cm (±1 mm) above the glass plate in the crystallisation chambers, in combination with 26°C (±1°C) in the outer chamber, thereby intending an average crystallization starting time of 12–14 h. In contrast to LBI, at BRAD no cooling apparatus was applied for regulating the temperature of the outer chamber for periods of atmospheric temperatures above 26°C. In both laboratories, the evaporation process was monitored by means of a digital camera. Based on the photographs obtained, evaporation time was determined for each biocrystallogram, that is, the time in hours/minutes from the onset of the experiment until the appearance of the first crystal.

### 2.8. Visual Evaluation

During the initial screening investigations of the eight coded preparations A–H, various morphological characteristics of the resulting biocrystallograms, including validated morphological criteria [12], as well as different types of overall structural criteria according to Selawry [16], were applied (not reported here). Also different visual evaluation techniques were examined, including grouping, nonparametric ranking, and parametric scoring (not reported here).

In the main experiments, visual evaluation according to validated morphological criteria [12] was initially planned, but could not be performed due to so-called peripheral crystallisations (see Section 3) that unexpectedly occurred in the biocrystallograms of one laboratory, due to an extended period of unusually high atmospheric temperature during the experiments of summer 2008.

### 2.9. Computerised Textural Image Analysis

The biocrystallograms were scanned after a minimum storage time of 48 hours applying a UMAX PowerLook III scanner with 256 grey levels. The RBG distribution was set to 33/33/33, and for preprocessing normalisation a Gaussian normalisation was applied. The applied texture image analysis technique was described by Carstensen [20] and concerning the specific application in a biocrystallisation context by Andersen et al. [21]. The 15 second-order parameters used to characterize the grey level cooccurrence matrices (GLCMs) were applied for analysis, in the following termed TA parameters. Out of the available 5 resolution scales only scale 1 was used. Circular regions of interest (ROI) were defined around the geometrical centre of the biocrystallograms, with a diameter *D*_*x*_ given as percentage of the total biocrystallogram diameter *D* (90 mm, set to 100%). Circular segments were defined as area differences between two ROIs. In this study, we primarily analyzed the entire biocrystallograms (ROI 0–100%) and additionally segments of 0–50%, 50–70%, 70–90%, and 90–100%.

In the screening experiments, texture analysis data were acquired but not statistically evaluated due to the restricted number of experiments. For the analysis of the main experiments, all scanned biocrystallograms of the 7 LBI and 8 BRAD experiments were used, minus those discarded due to technical errors (see below).

### 2.10. Missing Data

Five biocrystallograms (out of 15 × 36 = 540 in total) were discarded due to technical error, primarily due to leakage of solution from under the surrounding acrylic ring. Thus 535 biocrystallograms were used for computerized texture analysis.

### 2.11. Analysis of Variance (ANOVA)

Evaporation time and texture analysis data were analyzed by means of ANOVA *F*-tests with the independent parameters experimental day {1–15} (N° 1–7 correspond to experiments 1–7 from LBI and N° 8–15 to experiments 1–8 from BRAD), treatment {1-2} (*Stannum met.* 30x, water 30x), and internal replicate {1–3} using the software Statistica 4.1 for Macintosh (StatSoft Inc., Tulsa, USA). Since the investigation was exploratory in nature, no corrections for multiple testing were applied in general; in one evaluation, the Bonferroni-Holm correction for multiple testing was used.

## 3. Results

### 3.1. Seedling Weight

Seedling weight data points were defined as average weight of all collected seedlings per experimental treatment. Thus 6 data points (3 treatments B and G, resp.) were available per experiment. 2-way analysis of variance with the independent variables experimental day (1–7 and 1–8, resp.) and potency treatment (code B, G) and the dependent variable average weight was performed separately for the experiments performed at LBI and BRAD. No significant effects were detected (*F*-test of main effects and interaction >0.05 in any case). Thus, no differences in weight were observed in either laboratory due to the treatment with *Stannum met.* 30x compared to water 30x.

There was a small, but statistically significant (*P* < 0.001,  *t*-test), systematic difference in seedling weight between both laboratories: seedlings at BRAD exhibited on average a 2.7% larger weight compared to seedlings at LBI. This may be due to a slightly higher temperature in the BRAD heating cabinet.

### 3.2. Biocrystallisation Evaporation Time

**Figure 3:**
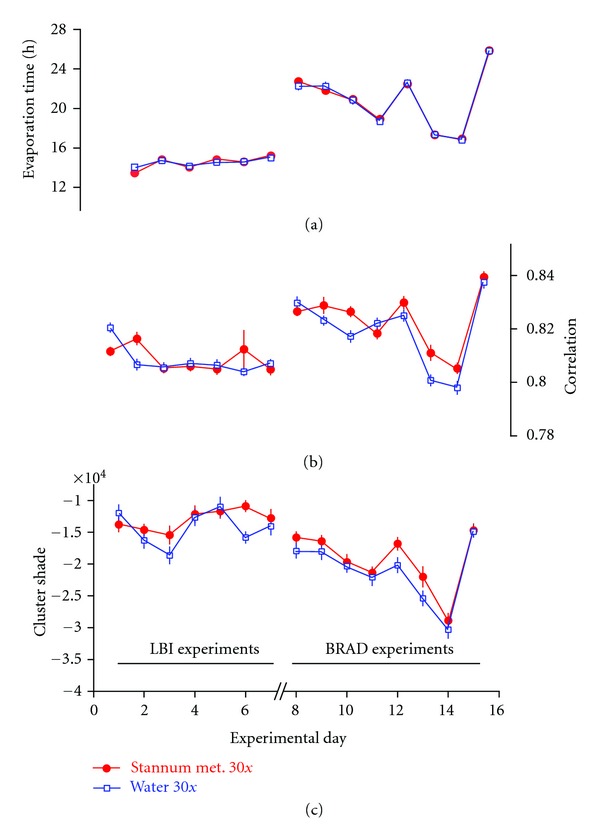
(a) Average evaporation times (mean ± SE of 6 replicates each) of the cress extract biocrystallograms obtained at the individual experimental days. Standard errors are smaller than the icons used and thus not visible. Data from day 1 are missing due to technical failure. ((b), (c)) Texture analysis variable (b) “correlation” (member of variable group II) and (c) “cluster_shade” (member of variable group I) (mean ± SE of 6 replicates each) for biocrystallograms of cress grown in either *Stannum met.* 30x or water 30x, plotted as a function of the individual experiments (experimental day). Connecting lines are no interpolations.

At LBI the average evaporation time for the biocrystallograms of the potency preparations B and G over the 7 experiments showed a range of about 13.5–15 h, that is, partly beyond the intended range of 12–14 h (Figure 3(a)). At BRAD average evaporation time showed a range of about 17–26 h, that is, markedly beyond the intended range, due to an extended period of unusually high atmospheric temperature during the 5 experiments of June/July 2008.

Based on analysis of variance (ANOVA), for both labs a highly significant effect of experimental day was found (*P* < 0.0001), whereas no differences (*P* > 0.48) were found for homeopathic treatment {preparation B/G}, nor for the internal replicates {1/2/3}, nor any interaction. Thus evaporation time did not differ between the different experimental treatments, though there were differences in absolute values from experiment to experiment (see Figure 3(a)).

### 3.3. Texture Analysis: Entire Biocrystallograms

Texture analysis (TA) data of scale 1 from the entire biocrystallograms (region of interest (ROI) 0–100%) were analyzed by full 3-way analysis of variance, performed on all 15 second-order TA variables, using experimental day {1–15}, treatment {*Stannum met.* 30x, water 30x}, and internal replicate treatment {1–3} as the three independent variables. Results are given in Table 1.

 All 15 second-order TA variables yielded significant or highly significant results for the potency treatment, meaning that a difference was found in the texture of biocrystallograms of cress seedlings germinated in Stannum met. 30x versus water 30x. No highly significant differences were observed between the three internal replicate treatments. However, two TA variables showed a significant internal replicate effect pointing towards a weak systematic error (for further analysis and discussion see below). Furthermore, there were significant differences in absolute values for all TA variables between the single experimental days (significant experimental day effect). A graphical representation of two TA variables is given in Figure 3.

12 out of 15 second-order TA variables yielded significant or highly significant interactions between experimental day and potency treatment (Table 1, Interaction 1-2), meaning that the difference between *Stannum met.* 30x and water 30x seemed to exhibit varying results for different experimental days. Three out of 15 TA variables did not show any such interaction meaning that the effects of *Stannum met.* 30x versus water 30x were quite reproducible over the 15 independent experiments for these three variables. Figure 3 depicts one example for each of both types of TA variables (time modulated and reproducible). Though at first glance there may seem to be only little difference between these two variables, the difference manifests in the number of quantitative effect inversions as a function of experimental day: time-modulated variables exhibit 5–7 numeric effect inversions, whilst variables without interaction (“cluster_shade,” “diagonal_moment,” “maximum_probability”) show only 2–4 numerical effect inversions. Only peripherally significant effects were detected for interactions involving the internal treatment replicates (Table 1, Interactions 1-3, 2-3, and 1-2-3).

Many of the 15 TA variables were correlated to each other, meaning that—in the present case of biocrystallograms and scale 1 analysis—most variables were not independent. Two main groups of variables can be distinguished, which were closely correlated within the respective group, but not or only weakly correlated to those of the other group:

- group I (“cluster_shade” and “diagonal_moment,” *r* = 0.96 within group),
- group II (all other TA variables with the exception of “cluster_prominence,” *r* > 0.6 within group).

The variable “cluster_prominence” showed a medium correlation to all but one other TA variables and takes an intermediate position. Variables of group II were correlated to evaporation time (*r* > 0.46, see also Figure 3(b) for a representative example), whilst variables of group I were not significantly correlated to evaporation time (see Figure 3(c) for an example). Variables of group II had in all but one case a significant interaction of potency treatment with experimental day, whilst variables of group I did not exhibit any such interaction.

The high correlation amongst the TA variables considerably complicates a conceptually coherent correction for multiple testing. We nevertheless applied the Bonferroni-Holm procedure to the statistical tests of Table 1 under the very conservative assumption of 105 independent statistical tests (7 tests × 15 2nd-order TA variables). Besides experimental day, 3 main potency effects and 6 potency/experimental day-interactions were significant at *P* < 0.05 (marked with asterisk in Table 1).

### 3.4. Texture Analysis: Sectors of Biocrystallograms

**Figure 4:**
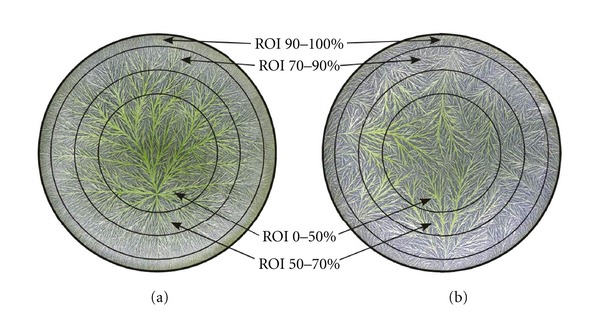
Typical biocrystallograms of watery extract of cress seedlings (310 mg extract 10% (w/v)/150 mg CuCl_2_/6.1 mL volume) for a crystallisation time of (a) 15 h and (b) 24 h. Prolonged crystallisation as in case (b) led to crystals growing from the periphery to the centre. ROI: circular regions of interest, defined by radius segments 0–50%, 50–70%, 70–90%, and 90–100%.

In a subgroup analysis we aimed at identifying the spatial region of the biocrystallogram where the potency treatment effect manifests predominantly. We defined four different ROIs as circular segments of the entire biocrystallogram by the radius segments 0–50%, 50–70%, 70–90%, and 90–100% (Figure 4). Full 3-way analysis of variance was performed on all 15 second-order TA variables, using experimental day {1–15}, treatment {*Stannum met.* 30x, water 30x}, and internal replicate treatment {1–3} as independent variables. Results for the potency treatment main effect and the interaction of potency treatment with experimental day are given in Table 2. The signal in the variables without interaction (group I, “cluster_shade” and “diagonal_moment”) manifests predominantly in the geometrical centre of the biocrystallogram (ROI 0–70%). For the TA variables showing interactions with experimental day (group II), there seem to be two signal sources: (i) a reproducible signal (main effect) rather in the periphery of the biocrystallograms (70–90%) and (ii) a signal varying in time (interaction with experimental day) spread all over the biocrystallogram with emphasis on ROI 50–70% (see Table 3 for a summary).

### 3.5. Texture Analysis: Prolonged Evaporation Time

Biocrystallograms with a prolonged evaporation time (>18–20 h) exhibited so-called peripheral crystallisations, that is, ramification structures, which expand from the periphery of the biocrystallogram towards the geometrical centre (Figure 4(b)). Hereby the peripheral ramifications interfere with the ramification structure observed in “optimal” 1-centred biocrystallograms, expanding from the centre zone to the periphery (Figure 4(a)). In the experiments at BRAD, average evaporation time showed a range of about 17–26 h, that is, markedly beyond the intended range of 12–14 h (Figure 3(a)), due to the lack of a cooling apparatus during an extended period of unusually high atmospheric temperature in summer 2008. We thus wondered whether there was any correlation between the effect of the homeopathic treatment and evaporation time, given the marked differences in biocrystallogram morphology for high evaporation times (Figure 4) and given the different spatial regions of the biocrystallograms yielding a homeopathic treatment signal. We therefore analyzed the correlations between the homeopathic treatment effects and the evaporation time for all 15 variables of texture analysis for all 8 experiments at BRAD; treatment effects were defined as mean numerical differences between the *Stannum met.* 30x and water 30x groups for all TA variables. Out of all 15 variables of texture analysis, only “sum_energy” exhibited a significant correlation (*r* = 0.839, *P* < 0.01): differences between *Stannum met.* 30x and water 30x were more pronounced for lower evaporation times. For all other 14 TA variables, there was no such correlation. However, visual inspection of the corresponding scatterplots showed a tendency for a correlation when excluding experiments no. 8 and 11 (showing effect inversions, see Figure 3(b)): the homeopathic effects seemed to be more pronounced for lower evaporation times.

### 3.6. Texture Analysis: Comparison between the Two Laboratories

A comparison between the results of the experiments at the two laboratories in Denmark (BRAD) and in The Netherlands (LBI) was effectuated in the frame of a 4-way ANOVA with the independent factors (1) laboratory {BRAD, LBI}, (2) experimental day {1–7}, (3) treatment {*Stannum met.* 30x, water 30x}, and (4) internal replicate {1–3}. In order to achieve a fully balanced experimental design, the 8th experiment at BRAD was omitted from this analysis. Dependent variables were all 15 TA variables, calculated based on the entire biocrystallogram area (ROI 0–100%). Regarding the independent variables treatment, experimental day, and internal replicate, results were essentially similar to the 3-way ANOVA presented above (Table 1). Any difference between the laboratories regarding the effect size of the homeopathic treatment should manifest in significant interactions between the factors laboratory and treatment. Five out of 15 TA variables displayed such interactions: “correlation” (*P* = 0.037), “inertia” (*P* = 0.040), “sum_energy” (*P* = 0.008), “sum_entropy” (*P* = 0.018), and “sum_variance” (*P* = 0.017). In all five cases, differences between the homeopathic treatment groups were significant in the BRAD experiments (*P* < 0.001, LSD test), but not at LBI (*P* > 0.05, LSD test). All these variables belong to group II (see Table 3). Numerically, the effects of the homeopathic treatment were in the LBI experiments between 10–30% of those at BRAD regarding these five TA variables. The other 10 TA variables did not show any interaction between the factors laboratory and treatment.

### 3.7. Texture Analysis: Internal Replicates

As can be seen in Table 1, two TA variables (“inverse_different_moment” and “kappa”) showed a significant internal replicate effect pointing towards a systematic error, and two further TA variables (“diagonal_moment” and “maximum_probability”) showed a borderline significance. The hypothesis arose that this might be due to a laboratory processing order effect, since all six cress samples of a given experiment (Figure 2) were extracted one after the other before being mixed with dihydrate CuCl_2_ and pipetted onto glass plates in the crystallisation chamber, thus leading to a systematically increasing time span between extraction and crystallisation for the six cress samples. We tested the hypothesis that the internal replicate effect observed is due to a laboratory processing order effect as follows: for all 15 TA variables, data from the water 30x groups were normalized to the corresponding mean value for each experimental day. Data were then pooled and analyzed with a 1-way ANOVA with the independent variable “processing order,” varying between 1 and 6. This analysis yielded significant effects for three TA variables: “inverse_different_moment” (*P* = 0.020), “kappa” (*P* = 0.019), and “maximum_probability” (*P* = 0.004). In all cases, there was an approximately linear trend in the data associated with processing order.

Thus the question arose whether this processing order effect might lead to false-positive results regarding the two homeopathic preparations in question. If the randomization applied led to a balanced distribution of both groups regarding processing order, any systematic processing order effect would cancel out. However, the manual randomization led to an uneven distribution: the *Stannum met.* 30x treated group had a higher mean processing order number than the water 30x group. We therefore tried to correct for processing order by calculation of a linear regression between processing order number and experimental outcome and by subtraction of this correlation from the measured data for the three TA variables: “inverse_different_moment,” “kappa,” and “maximum_probability”. In Table 4, we compare the results of the uncorrected and corrected data for these three variables. The correction successfully eliminates the main internal replicate effect and leads to a somewhat weaker effect of the homeopathic treatment, albeit still significant. The effect interactions remain essentially unchanged.

## 4. Discussion

### 4.1. Biocrystallisation and Texture Analysis

We investigated the effects of a treatment with either *Stannum metallicum* 30x or water 30x on growing cress seedlings using the biocrystallisation method. In a series of 15 independent experiments, evaluation of the resulting biocrystallograms with computerized texture analysis yielded highly significant differences between the two homeopathically treated groups. Experiments were performed independently in two laboratories in Denmark and in The Netherlands and were fully randomized and coded (blinded).

Computerized texture analysis was performed with 15 second-order variables used to characterize the grey level co-occurrence matrices (GLCMs). In the present case, two essentially independent groups of texture analysis variables could be identified. Group I consisted of two closely correlated variables (“cluster_shade” and “diagonal_moment”) and showed a reproducible effect of the homeopathic treatment over all experiments in both laboratories (significant main effects and no interactions in the ANOVA model). The corresponding signal was located in the centre (ROI 0–70%) of the biocrystallograms. Group II consisted of 12 closely correlated TA variables that showed a significant main effect of the homeopathic treatment in the analysis of variance, but exhibited also a significant interaction with experimental day and partially also between the two laboratories. This means that the effect of homeopathic potencies was modulated by a—still unknown—factor associated with the experimental day and/or the laboratories. In contrast to the first group of variables, the reproducible part of the signal was located in the periphery of the biocrystallograms (ROI 70–90%). The variable part of the signal was located rather in the centre of the biocrystallograms (50–70%), but to some extent also spread all over the biocrystallogram.

The TA variables of group I (“cluster_shade” and “diagonal_moment”) showed a more stable response towards the homeopathic treatment, compared to variables of group II. The latter variables not only exhibited varying effects as a function of time (highly significant interaction between treatment and experimental day), but also showed larger differences in effect size between the two laboratories. Furthermore, the processing order effect manifested only in variables of group II. Thus the variables “cluster_shade” and “diagonal_moment” seem to be of primary interest as outcome parameters for follow-up studies, showing a reproducible (regarding experimental days as well as the two laboratories) and highly significant (*P* < 0.001) effect of the homeopathic treatment over all experiments. Additionally, a restriction of the analysis to the centre of the biocrystallogram (ROI 0–70%) seems to further increase the differentiation between the homeopathically treated groups.

The main statistical model applied was a classical analysis of variance with the independent factors experimental day (1–15), treatment (*Stannum met.* 30x, water 30x), and internal replicate (1–3). Repeated measures ANOVA (e.g., regarding experimental day) was assumed to be inappropriate because measurements at different days were performed on different subjects (seedlings and biocrystallograms). Discriminant analysis was not used because we wanted to investigate possible interactions of treatment with experimental day and internal replicate, as well as main effects of the internal replicates. Statistical models used for future experiments could include evaporation time as continuous covariate. Since the investigation was exploratory in nature, no corrections for multiple testing were applied in general. Furthermore, the high correlation amongst the TA variables considerably complicates a conceptually coherent correction for multiple testing. We nevertheless applied the Bonferroni-Holm procedure to the statistical tests of Table 1 under the very conservative assumption of 105 independent statistical tests (7 tests × 15 2nd order TA variables). Besides experimental day, 3 main potency effects and 6 potency/experimental day interactions were significant at *P* < 0.05 (marked with asterisk in Table 1). Future experiments should include full systematic negative control experiments to test the appropriateness of the statistical model used, in order to avoid false-positive as well as false-negative results.

One may wonder whether the differences identified by texture analysis can be identified by human visual inspection. In the framework of the Triangle cooperation, 14 visual evaluation criteria have been defined and validated according to ISO 11035 [12]. These visual criteria and the variables of texture analysis had been compared in detail, but no reproducible correlation between these two sets of variables could be identified [13]. In our present investigation, the optical impression was that the differences between the biocrystallisation replicates were larger than the differences between the differently treated groups. Furthermore, application of the Triangle visual evaluation criteria is hampered for the BRAD biocrystallograms due to the frequent occurrence of peripheral crystallisations (Figure 4(b)). We nevertheless hope to identify visual correlates to texture analysis features in the context of a forthcoming investigation.

### 4.2. Interpretation of Biocrystallograms

Can any conclusions be drawn regarding some characteristics or properties of the differently treated seedlings, based on the observations in texture analysis parameters? In other words, is there a straightforward interpretation of biocrystallograms? The biocrystallisation method originally was developed to complement classical analytical procedures that aim at identifying quantifiable amounts of defined inorganic or organic compounds. The complementary scope of biocrystallisation has been to characterize *qualities* and *formative forces* of agricultural and horticultural crop samples as well as milk or plant extracts used for remedy production in anthroposophic pharmacy [3]. Further applications were developed in medicine, where blood biocrystallograms have been used for early diagnosis of certain illnesses [4, 22, 23]. Whilst we do not know clinical trials that focused on the use of blood biocrystallisation for diagnostic purposes, the investigation of agricultural and horticultural crop samples was successful in differentiating products from organic and conventional farming systems in single trials [8, 11, 24, 25]—a differentiation that is difficult to achieve by analytical methods only [26, 27].

In most agricultural applications, biocrystallisation has been used as a descriptive, phenomenological, and comparative method. The exact physicochemical processes governing the formation of biocrystallograms are not well known and still under discussion [28]. For a successful application in an agricultural context [8–11], the exact knowledge of these processes does not seem to be of ultimate importance, however. Some scientists working with biocrystallisation compare the evaluation of biocrystallograms with the understanding and the interpretation of pictures in a broader sense [29, 30]. In such an approach, the content of the picture is described in qualitative terms, meaning that the content and/or the interpretation are more important than the materials that were used to paint the picture (e.g., canvas, colours, etc.). In other words, the focus is on the information rather than on the information carrier.

In our investigation, we used texture analysis as outcome measure. We identified differences between the homeopathically treated groups and concluded that there were some specific biological effects of *Stannum met.* 30x on the germinating cress seedlings, compared to water 30x as control. An interpretation of these effects is difficult at the present stage of investigation, however. Inferences on specific changes in material composition of the cress seedlings were not possible, also due to the lack of knowledge of the physicochemical details of the biocrystallisation method. An indirect qualitative interpretation of the texture analysis data is not yet possible due to missing comparable investigations. And the still too large variability of the biocrystallisation replicates hampers a comparative qualitative interpretation by means of validated visual criteria. Analogously, a direct informational interpretation of the “meaning” of the biocrystallograms is not possible at the moment. However, the differences identified by texture analysis are highly significant, revealing the potential of this method and calling for follow-up investigations enabling researchers to interpret the effects found. We attribute a considerable potential to the biocrystallisation method, since it combines quantitative and qualitative aspects, which we think to be necessary for the analysis of complex systems, where classical analytical methods are too limited to allow a thorough characterization.

### 4.3. Methodical Strengths and Weaknesses

The experimental design involved three independent replicates for each homeopathic preparation investigated. Starting from one common 28x preparation of *Stannum met.* and water each, three *Stannum met.* 30x potencies and three water 30x control samples were independently produced and packed in duplicate for each of the 15 single experiments. The six 30x samples were randomized and coded (blinded) for each experiment, and each sample was independently assessed with the cress bioassay and six subsequent biocrystallogram replicates per sample. The threefold sample replicate per experiment was used to estimate the stability of the experimental set-up by inclusion in the analysis of variance as independent factor (internal replicate 1/2/3). In the analysis, we observed significant main effects of the internal replicate for two texture analysis variables. Since the internal replicate was not randomized in itself, the order of the internal replicates essentially reflects the processing order of the samples in the laboratories. We thus investigated a possible influence of processing order on experimental outcome by an analysis of the water 30x control samples of all experiments and identified a significant processing order effect for three texture analysis variables. In a control calculation we subtracted a linear estimation of the processing order effect from the data, which eliminated the internal replicate effect. Due to an unbalanced distribution of the two treatments regarding processing order, the homeopathic treatment effect was somewhat weaker (albeit still significant) after this correction. Two main conclusions can be drawn from this evaluation for future experiments: (1) the processing order effect should be minimized or eliminated by a modification of the experimental procedure; (2) randomization of samples should be accomplished by computer-generated randomization lists leading to an overall balanced distribution of the investigated parameters regarding processing order.

*Stannum met.* 30x is an ultramolecular preparation. The metal is triturated in the solid phase in lactose up to the potency level 4x and from there on potentised in the liquid phase in distilled water up to 30x. Correspondingly, *Stannum met.* 30x contains tin from the starting material in a nominal dilution of 10^−30^ (8.4 × 10^−30^ M) and lactose at a nominal dilution of 10^−24^ (2.9 × 10^−24^ M). This preparation was compared to water 30x, that is, water that has undergone 30 analogous steps of dilution and agitation. Thus, the two preparations were exactly comparable in terms of physicochemical treatment for the last 24 dilution steps, meaning that the differences found have to be traced back to the substances potentised: *Stannum met.* 30x and/or lactose 24x. According to clinical homeopathic experience, lactose can be regarded as quite neutral carrier that does not induce considerable therapeutic effects. Whether this is also true in the present case of the biocrystallisation of cress cannot be decided yet. In order to allow a definite statement that the effects found can be attributed to the metal potentised, another type of experimental control should be used in forthcoming investigations, either another homeopathic metal preparation 30x or lactose 30x.

The homeopathic preparations were not produced under sterile conditions. Since the potentisation medium was not a water-ethanol mixture but distilled water, microbial contamination cannot be excluded a priori. However, since a high number of independently produced samples was used (*n* = 90), a single contamination would not lead to statistically significant effects. Contamination of multiple samples would be expected to be randomly distributed on the two experimental conditions and hence most probably should not lead to false-positive results.

Reactivity towards homeopathic preparations of a given harvest lot may depend on the age and the lot-specific cultivation and harvest history, as has been observed for the barley/gibberellic acid model [31] and the dwarf pea/gibberellic acid model [32]. A comparison of different seed lots with contrasting characteristics (e.g., size, density, age, vigour level) regarding their response towards the homeopathic treatment will not only help in optimizing the power of the bioassay, but will also be necessary to investigate applicability in replication trials.

Since this study was exploratory in nature, we did not perform full systematic negative control experiments, but chose to determine experimental stability by the analysis of the internal replicates. The use of full systematic negative control experiments in future investigations will not only allow an even more thorough test of experimental stability, but also allow to empirically determine most appropriate statistical models.

Summarising, methodical strengths of our study are the parallel investigations in two independent laboratories, the rather high number of independent experiments and internal replicates, the randomization and blinding procedures applied, the objective outcome measure, and the investigation of the internal stability of the experimental set-up by the use and statistical analysis of the internal replicates. Methodical weaknesses are the—at the moment—lacking interpretation of the differences found, the nonsterile preparation of the homeopathic samples, the processing order effect, the lack of full systematic negative control experiments, and the excess in evaporation time in one laboratory leading to unwanted peripheral crystallisations. None of these weaknesses should lead to false-positive results, however.

### 4.4. Comparable Investigations

According to our knowledge, Selawry [16] was the first and only scientist who published a comparable investigation. She germinated various plants (oats, peas, beans) in different metal potencies (silver, lead, tin, copper, iron, etc.), mostly in two potentisation levels (6x and 30x) and produced biocrystallograms from extracts of the seedlings. She described the results obtained by qualitative sketches of typical biocrystallograms. No statistical analysis was performed on groupings of biocrystallograms, or ratings of certain features, or quantitative image analysis, which was not easily available at that time. Due to the different evaluation approaches, laboratory techniques, and so forth a closer comparison of her and our results is not feasible.

As discussed, one group of texture analysis variables (“cluster_shade” and “diagonal_moment”) showed a reproducible (regarding experimental days as well as the two laboratories) and significant effect of the homeopathic treatment over all 15 experiments, whilst group II of texture analysis variables exhibited an interaction of the potency effect with experimental day and partially also with the laboratories involved. We know only a few homeopathic basic research investigations that used a comparable number of independent experiments [33–37], but to the best of our knowledge only Pelikan and Unger [35] and Brizzi et al. [37] assessed internal reproducibility by statistical means. Pelikan and Unger observed internally reproducible effects of silver nitrate potencies on wheat growth, whilst Brizzi et al. observed an effect of arsenic trioxide potencies on wheat germination that increased with time. In the light of the considerable problems to achieve independent laboratory-external reproductions of homeopathic basic research models [1, 38], observations of internally reproducible effects are important to exclude the hypothesis of an inherent irreproducibility of effects of homeopathic preparations due to the nature of their mode of action [1].

### 4.5. Conclusions and Outlook

Using computerized GLCM texture analysis of biocrystallograms, we observed significant effects of a homeopathic treatment with *Stannum metallicum* 30x compared to water 30x on germinating cress seed over 15 independent experiments in two independent laboratories. Showing a reproducible (regarding experimental days as well as the two laboratories) and highly significant effect of the homeopathic treatment, two variables of texture analysis (“cluster_shade” and “diagonal_moment”) seem to be of primary interest for follow-up studies.

It will be interesting to analyse the biocrystallograms of the present trial in more detail. Investigations with texture analysis in other resolution scales will provide information regarding the spatial localisation of the differences in texture. Other approaches of image analysis (e.g., fractal dimension) may add complementary information helping to further describe and understand the effects found. Interpretation of the biocrystallogram features identified will need additional investigations. Methodical improvements are needed to eliminate the processing order effect. Furthermore, a comparison of different seed lots with contrasting characteristics (e.g., size, density, age, vigour level) regarding their response towards the homeopathic treatment is necessary.

Even though interpretation of the biocrystallograms is still open, it seems interesting to us applying the experimental set-up developed to open questions of homeopathic basic research. This may concern specificity (are there any differences in effects between different homeopathic preparations?), stability against external influences (e.g., light, heat, electromagnetic radiation) as well as the mode of action of homeopathic preparations. In addition, a successful formal repetition study with a predefined primary outcome measure may be considered as strong argument for specific properties of homeopathic preparations. Such a formal repetition study should incorporate full systematic negative control experiments in order to ensure the stability of the experimental set-up and the appropriateness of the statistical model applied.

We think that the combination of the biocrystallisation method with other established outcome measures (such as seedling weight and length) will provide additional valuable information. We assume that the biocrystallisation method will develop into a valuable complementary evaluation method for any plant model in homeopathic basic research.

## Figures and Tables

**Table 1:** Results from ANOVA *F*-tests for all 15 second-order variables of texture analysis (main effects and interactions).

Texture analysis parameter	Main effects (*P* value)			Effect interactions (*P* value)			
	(1) Exp. day	(2) Potency	(3) Replicate	1-2	1-3	2-3	1-2-3
Cluster_prominence	<0.0001*	0.0436	0.2143	**0.0005**	0.1135	0.3126	0.1706
Cluster_shade	<0.0001*	0.0004*	0.1296	0.3264	0.2145	0.4365	0.5069
Correlation	<0.0001*	**0.0039**	0.5376	0.0001*	0.1567	0.0918	0.1896
Diagonal_moment	<0.0001*	0.0002*	0.0508	0.2998	0.0943	0.6176	0.3498
Difference_energy	<0.0001*	**0.0076**	0.3948	**0.0040**	0.2444	0.1625	0.1077
Difference_entropy	<0.0001*	**0.0059**	0.5062	**0.0009**	0.2248	0.1288	0.1592
Energy	<0.0001*	**0.0055**	0.4246	0.0143	0.3051	0.1684	0.1437
Entropy	<0.0001*	0.0150	0.2997	**0.0006**	0.1970	0.1947	0.1318
Inertia	<0.0001*	**0.0041**	0.5262	0.0001*	0.1567	0.0978	0.1934
Inverse_different_moment	<0.0001*	**0.0024**	0.0166	0.0001*	0.0893	0.2193	0.0138
Kappa	<0.0001*	**0.0023**	0.0112	<0.0001*	0.0923	0.2521	0.0124
Maximum_probability	<0.0001*	**0.0022**	0.0528	0.2930	0.1958	0.1342	0.0886
Sum_energy	<0.0001*	0.0002*	0.4163	**0.0046**	0.0902	0.0397	0.3965
Sum_entropy	<0.0001*	**0.0013**	0.7932	0.0001*	0.1380	0.0387	0.1780
Sum_variance	<0.0001*	**0.0030**	0.6470	<0.0001*	0.1622	0.0507	0.1598

*Note.* Independent experimental parameters were (1) experimental day {LBI: 1–7, BRAD: 8–15; *n* = 15}, (2) treatment {*Stannum met.* 30x or water 30x; *n* = 2}, and (3) internal treatment replicate {1–3; *n* = 3}. Each experimental parameter combination (statistical treatment cell) was assessed by 6 biocrystallogram replicates. Highly significant effects (*P* < 0.01) are shown in bold. Statistical tests significant at *P* < 0.05 after a Bonferroni-Holm correction are marked with an asterisk.

**Table 2:** Results from ANOVA *F*-tests for the potency treatment (main effect and interaction with experimental day) in four different regions of interest (ROI).

Texture analysis	Main effects (*P* value)				Interaction with exp. day (*P* value)			
parameter	ROI 0–50%	ROI 50–70%	ROI 70–90%	ROI 90–100%	ROI 0–50%	ROI 50–70%	ROI 70–90%	ROI 90–100%
Cluster_prominence	0.6344	0.3447	**<0.0001**	0.0651	0.1557	0.0175	**0.0085**	0.0493
Cluster_shade	**0.0038**	**0.0002**	0.1552	0.1584	0.6700	0.9604	0.8339	0.9768
Correlation	0.6482	0.0938	**<0.0001**	0.0131	0.0258	**<0.0001**	0.0186	0.1024
Diagonal_moment	**0.0021**	**<0.0001**	0.1406	0.0764	0.4649	0.9091	0.8331	0.9135
Difference_energy	0.6773	0.0160	**<0.0001**	0.0131	0.0217	**0.0001**	0.0380	0.0419
Difference_entropy	0.8924	0.0461	**<0.0001**	0.0123	0.0186	**<0.0001**	0.0240	0.0570
Energy	0.7202	0.0170	**<0.0001**	0.0235	0.0292	**0.0001**	0.0330	0.0467
Entropy	0.9311	0.0578	**<0.0001**	0.0416	0.0207	**<0.0001**	0.0172	0.0351
Inertia	0.6508	0.0939	**<0.0001**	0.0145	0.0258	**<0.0001**	0.0186	0.1158
Inverse_diff moment	0.3531	0.0199	**<0.0001**	**0.0069**	0.0103	**<0.0001**	0.0379	**0.0096**
Kappa	0.3137	0.0339	**<0.0001**	**0.0085**	0.0120	**<0.0001**	0.0271	**0.0083**
Maximum_probability	0.4692	0.1979	0.0140	0.0653	0.1840	**0.0091**	0.3755	0.0246
Sum_energy	0.1270	0.0550	**<0.0001**	**0.0008**	0.0129	**<0.0001**	0.0603	0.0498
Sum_entropy	0.5550	0.1241	**<0.0001**	**0.0026**	0.0280	**<0.0001**	0.0289	0.0415
Sum_variance	0.6193	0.0926	**<0.0001**	**0.0071**	0.0260	**<0.0001**	0.0180	0.0487

*Note.* Results from ANOVA *F*-tests for the potency treatment (*Stannum met.* 30x versus water 30x, main effect and interaction with experimental day), analysing all 15 second-order variables of texture analysis in four different regions-of-interest (ROI, defined as circular segments in % radius). Results originate from a full 3-way ANOVA with the independent experimental parameters (1) experimental day {LBI: 1–7, BRAD: 8–15; *n* = 15}, (2) treatment {*Stannum met.* 30x or water 30x; *n* = 2}, and (3) internal treatment replicate {1–3; *n* = 3}. Each experimental parameter combination (statistical treatment cell) was assessed by 6 biocrystallogram replicates. Highly significant effects (*P* < 0.01) are shown in bold.

**Table 3:** Summary of the homeopathic potency effects for the two texture analysis variable groups.

Texture analysis variables group	I	II
Correlation to evaporation time	No	Yes
Reproducible (main) potency effect	Yes	Yes
Reproducible (main) potency effect location (ROI)	0–70%	70–90%
Variable potency effect (interaction with experimental day)	No	Yes
Variable potency effect (interaction with experimental day) location (ROI)	—	50–70%
Differences between the laboratories	No	Partially

*Note.* Texture analysis variable groups I: “cluster_shade” and “diagonal_moment”; II: all other TA variables except “cluster_prominence”.

**Table 4:** Results from ANOVA *F*-tests (main effects and 2nd-order interactions) for three variables of texture analysis, uncorrected and corrected for processing order.

Texture analysis parameter	Main effects					
	(1) Exp. day	(1) Exp. day corrected	(2) Potency	(2) Potency corrected	(3) Int. Replicate	(3) Int. Replicate corrected
Inverse_different_moment	**<0.0001**	**0.0001**	**0.0024**	0.0248	0.0166	0.9406
Kappa	**<0.0001**	**0.0001**	**0.0023**	0.0222	0.0112	0.9251
Maximum_probability	**<0.0001**	0.2074	**0.0022**	0.0147	0.0528	0.9981

Texture analysis parameter	Effect interactions					
	1-2	1-2 corrected	1-3	1-3 corrected	2-3	2-3 corrected

Inverse_different_moment	**0.0001**	**0.0001**	0.0893	0.0523	0.2193	0.3688
Kappa	**<0.0001**	**0.0001**	0.0923	0.0575	0.2521	0.4034
Maximum_probability	0.2930	0.2436	0.1958	0.1652	0.1342	0.2543

*Note.* Independent experimental parameters were (1) experimental day {LBI: 1–7, BRAD: 8–15; *n* = 15}, (2) treatment {*Stannum met.* 30x or water 30x; *n* = 2}, and (3) internal treatment replicate {1–3; *n* = 3}. Each experimental parameter combination (statistical treatment cell) was assessed by 6 biocrystallogram replicates. Highly significant effects (*P* < 0.01) are printed in bold.
